# Effects of cue validity on attentional selection

**DOI:** 10.1167/jov.22.8.15

**Published:** 2022-07-26

**Authors:** Hao Lou, Monicque M. Lorist, Karin S. Pilz

**Affiliations:** 1 Department of Experimental Psychology, University of Groningen, Groningen, the Netherlands; 2 Department of Experimental Psychology, University of Groningen, Groningen, the Netherlands; 3 Department of Biomedical Sciences of Cells and Systems, Cognitive Neuroscience Center, University of Groningen, Groningen, the Netherlands; 4 Department of Experimental Psychology, University of Groningen, Groningen, the Netherlands; 5 Cito Institute for Test Development, Arnhem, the Netherlands

**Keywords:** space-based attention, object-based attention, individual differences, cue validity, bootstrapping

## Abstract

Visual attention can be allocated to locations or objects, leading to enhanced processing of information at the specific location (space-based effects) or specific object (object-based effects). Previous studies have observed object-based effects to be smaller and less robust than space-based effects, with large individual differences in their temporal occurrence. Studies on space- and object-based effects are often based on a two-rectangle paradigm in which targets appear at cued locations more often than uncued locations. It is, however, unclear whether and how the target's spatial probability affects the temporal occurrence of these effects. In three experiments with different cue validities (80%, 50% and 33%), we systematically changed the interval between the cue and the target from 50 to 600 ms. On a group level and for individuals, we examined how cue validity affects the occurrence of object- and space-based effects. We observed that the magnitude and the prevalence of space-based effects heavily decreased with reduced cue validity. Object-based effects became even more sparse and turned increasingly negative with decreasing cue validity, representing a different-object rather than a same-object advantage. These findings indicate that changes in cue-validity affect both space- and object-based effects, but it does not account for the low prevalence and magnitude of object-based effects.

## Introduction

Visual attention is a selective process that focuses on specific locations (space-based attention) or objects (object-based attention) while ignoring other locations or objects. Space- and object-based attentional effects have been extensively investigated and demonstrated in previous studies (Al-Janabi & Greenberg, 2016; Duncan, 1984; Egly, Driver, & Rafal, 1994; Eriksen & Eriksen, 1974; Moore, Yantis, & Vaughan, 1998; Nah & Shomstein, 2020; Pilz, Roggeveen, Creighton, Bennett, & Sekuler, 2012; Posner, 1980; Posner, Snyder, & Davidson, 1980; Shomstein & Behrmann, 2008). Whereas space-based effects are strong and robust, object-based effects have been found to be smaller (Egly et al., 1994; Hecht & Vecera, 2007; Moore et al., 1998; Pilz et al., 2012) and prone to large individual differences (Pilz et al., 2012; Lou et al., 2020). Some studies even failed to find significant object-based effects (e.g., Macquistan, 1997; Shomstein & Behrmann, 2008).

The discrepancy in the prevalence of space- and object-based effects may relate to two factors: individual differences in the temporal occurrence of attentional selection and an imbalanced target distribution that favors the cued location. First, studies on space- and object-based effects usually employ a constant cue-to-target interval (e.g., Al-Janabi & Greenberg, 2016; Moore et al., 1998; Nah, Neppi-Modona, Strother, Behrmann, & Shomstein, 2018; Pilz et al., 2012). However, attention seems to be a rhythmic process that fluctuates with a periodicity in the theta frequency range (Drewes & VanRullen, 2011; Vanrullen, 2013; VanRullen & Koch, 2003), and it has been found that the presence of object-based effects varies with the duration of the cue-to-target interval (Fiebelkorn, Saalmann, & Kastner, 2013; Helfrich, Fiebelkorn, Szczepanski, Lin, Parvizi, Knight, & Kastner, 2018; Peters, Kaiser, Rahm, & Bledowski, 2021). It was also found that the temporal occurrence of object-based effects varied between individuals. Lou et al. (2020) recently found that object-based effects were observed at different cue-to-target intervals for different individuals. The large individual differences in the temporal occurrence of object-based effects might explain why object-based effects were small and even absent in previous studies with a fixed cue-to-target interval.

Second, an imbalanced target distribution might favor the cued locations. For example, in the commonly used two-rectangle paradigm examining space- and object-based effects, targets are present on the cued location in 70% to 80% of all trials (Egly et al., 1994; Fiebelkorn et al., 2013; Moore et al., 1998; Pilz et al., 2012). The remaining 20% to 30% of trials are split between the uncued locations, or contain catch trials in which no target is presented. Probability information has been shown to effectively guide attention (Druker & Anderson, 2010; Geng & Behrmann, 2005; Jiang, Swallow, & Rosenbaum, 2013; Kabata & Matsumoto, 2012). It is questionable that participants adopt a strategy that produces object-based cueing effects if there is no strategic advantage to doing so (Goldsmith & Yeari, 2003; Pilz et al., 2012). Therefore high cue validity may prompt attentional allocation to the cued location, leading to strong space-based effects. At the same time, targets are equally present on the two cued locations with a low probability (e.g., 10%). As participants respond more quickly or more accurately to spatial cues than to object-based cues (Egly et al., 1994; Hein, Blaschke, & Rolke, 2017; Moore et al., 1998; Pilz et al., 2012), spatial representations seem to play a more important role in guiding attention than object representations. This effect may account for the small or nonsignificant object-based effects observed in studies using the two-rectangle paradigm (Macquistan, 1997; Moore et al., 1998; Nah et al., 2018; Pilz et al., 2012; Lou et al., 2020).

In the current study, we further investigate whether and how probability of target appearance affects the temporal occurrence of attentional selection, and whether it affects the prevalence and magnitude of object-based effects, in particular. In three experiments, we employed a visual discrimination task combined with the two-rectangle paradigm (Moore et al., 1998; Pilz et al., 2012; Lou et al., 2020). We systematically changed the interval between the cue and target from 50 to 600 ms to measure temporal occurrence of space- and object-based attention, as in Lou et al. (2020). We manipulated cue validity among three experiments to further investigate the effects of probability of target appearance on attentional selection. Experiment 1 is comparable to previous studies using a similar paradigm with an 80% cue validity (Moore et al., 1999; Pilz et al., 2012; Lou et al., 2020). In Experiment 2, cue validity was decreased to 50%, and the remaining 50% of trials were distributed evenly between the two uncued locations. In Experiment 3, the probability of target appearance was equal for all three target locations. In all three experiments, we measured space- and object-based effects on group level and for individual participants. If probability information affects attention toward locations with the highest target probability, with decreased cue validity, attention would be less guided to the cued locations but more to the uncued locations within the cued object. Therefore we expect to find space-based effects to be smaller and less prevalent with decreased cue validity, and object-based effects to be stronger and more prevalent.

## Methods

### Participants

A total of 181 participants were recruited via Prolific (Palan & Schitter, 2018). The power analysis was conducted using G*Power based on the effect size of previous studies (Lou et al., 2020; Nah & Shomstein, 2020). Fewer participants are required to detect space- and object-based effects on the group level. Because we were also interested in individual variations in space- and object-based effects, we decided to follow the previous study (Lou et al., 2020) and set the target sample size at 40 participants. The study was preregistered on the Open Science Framework (https://osf.io/e8v2g). The data of 31 participants from Experiment 1, 20 participants from Experiment 2, and nine participants from Experiment 3 with low accuracy (overall accuracy below 60% or accuracy for each condition below 50%) were excluded from further analysis. The final sample comprised data from 121 participants (Experiment 1: *n* = 40, 18–25 years, *M* = 21.3, *SD* = 2.2, 23 males; Experiment 2: *n* = 41, 18–25 years, *M* = 20.4, *SD* = 2.2, 29 males; Experiment 3: *n* = 40, 18–24 years, *M* = 20.7, *SD* = 1.7, 30 males). All participants had normal or corrected-to-normal visual acuity. The Ethical Committee Psychology of the University of Groningen approved all experiments and procedures. Each participant only took part in one of the experiments. All participants gave informed consent and received monetary compensation for their participation.

### Apparatus, stimuli and procedure

**Figure 1.**
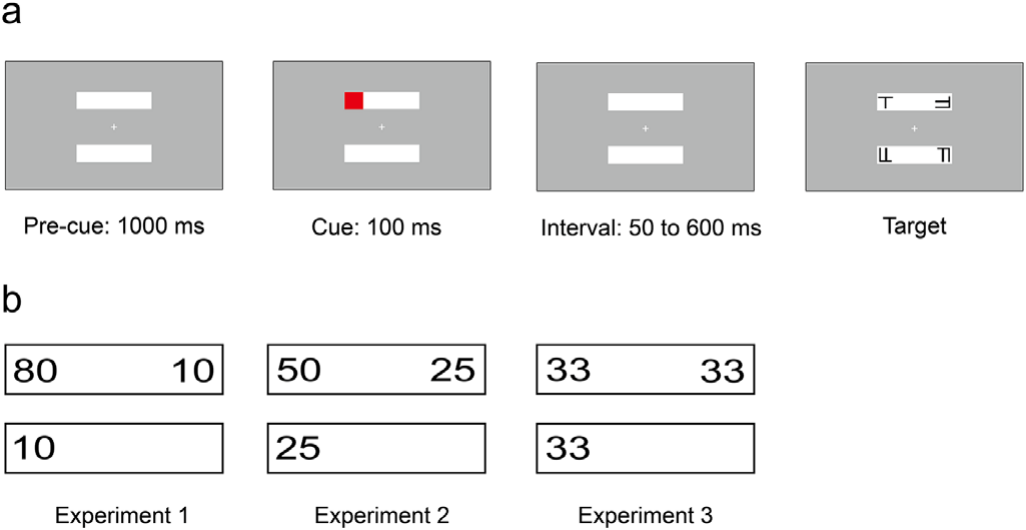
(a) Schematic diagram of trial sequence for a valid trial. (b) Probability of target appearance when cue appeared at the red cue position for each experiment. Location of the cue was random, but equally distributed across the four possible locations.

All experiments were created using PsychoPy/PsychoJS, v2020.1.3 (Peirce, Gray, Simpson, MacAskill, Höchenberger, Sogo, Kastman, & Lindeløv, 2019) and were conducted online using Pavlovia (www.pavlovia.org). The stimulus display consisted of two white rectangles on a gray background with a white fixation cross located in the center of the screen. The two rectangles were oriented horizontally and situated above and below the fixation cross^1^ (Figure 1a).

Each trial started with two rectangles presented for 1000 ms on the screen. Then a red square cue was presented for 100 ms in one end of the rectangles. After a randomly sampled 50 to 600 ms cue-to-target interval, a target letter “T” or “L” and three T/L hybrid distractors appeared at the four ends of the rectangles. Participants were asked to identify whether the target was a “T” or an “L” by pressing the “m” key (for Ts) or the “v” key (for Ls) on the keyboard as quickly and accurately as possible. The target could appear at one of three locations: the cued location (*valid trials*), the opposite end of the cued rectangle (*invalid-same trials*), or the same end of the uncued rectangle (*invalid-different trials*). The probability of targets appearing at these three locations varied between Experiment 1, 2 and 3 (Figure 1b). In Experiment 1, there were 1360 trials in total, of which 80% were valid trials, 10% were invalid-same trials, and 10% were invalid-different trials. In Experiment 2, there were 816 trials in total, 50% were valid trials, 25% were invalid-same trials, and 25% were invalid-different trials. In Experiment 3, targets appeared in three locations with an equal probability of 33% (272 trials each, 816 trials in total). A practice block of 15 trials preceded all experiments to familiarize participants with the experimental procedure.

### Analysis

Response times (RTs) faster than 150 ms, longer than 2000 ms, and beyond ±3 SD from the mean were removed from the analysis. Incorrect responses were also removed from the analysis. In total, 10.51% of the data were excluded from the analysis.

We calculated mean RTs for valid, invalid-same and invalid-different trials within 31 bins of 50 ms to assess space- and object-based effects for all cue-to-target intervals ranging from 50 to 600 ms. The first 50 ms bin comprised all cue-to-target intervals between 50 to 100 ms. Bins systematically shifted forward by 16.66 ms, ending with a bin comprising all cue-to-target intervals between 550 to 600 ms.

Overall space-based effects were calculated by subtracting mean valid RTs from mean invalid RTs for each cue-to-target interval. Overall object-based effects were calculated by subtracting mean invalid-same RTs from mean invalid-different RTs for each cue-to-target interval. A 3 (valid, invalid-same, invalid-different) × 31 (cue-to-target interval) repeated measure analysis of variance (ANOVA) was used to assess overall RT differences. Further false discovery rate (FDR) corrected *t*-tests were used to examine whether space- and object-based effects significantly greater than zero for each cue-to-target interval. In addition, we investigated whether the magnitudes of space- and object-based effects differ between experiments. We conducted a one-way ANOVA to space- and object-based effects separately with experiment as a between-subject factor. The *p* values were corrected for multiple comparisons using the FDR procedures (Benjamini & Hochberg, 1995).

The percentile bootstrap method (Efron, 1992; Efron & Tibshirani, 1993; Mooney Mooney, Mooney, Mooney, Duval, & Duvall, 1993; Pilz et al., 2012) was used to investigate individual differences in space- and object-based effects. For each participant, 999 bootstrapped data sets were constructed by resampling the original data randomly with replacement. Bootstrapped data sets contained the same number of trials in each condition as the original data, and were analyzed like the original sample. Space-based effects were calculated by subtracting the mean bootstrapped RTs of valid trials from the mean bootstrapped RTs of invalid trials. Object-based effects were calculated by subtracting the mean bootstrapped RTs of invalid-same trials from those of invalid-different trials. The 2.5 and 97.5 percentiles of the bootstrapped cueing effects were used to estimate the 95% confidence intervals for space- and object-based effects for each cue-to-target interval. Because of the high accuracy levels of our participants in all validity conditions, analyses of individual differences were only based on reaction times.

### Results

**Figure 2.**
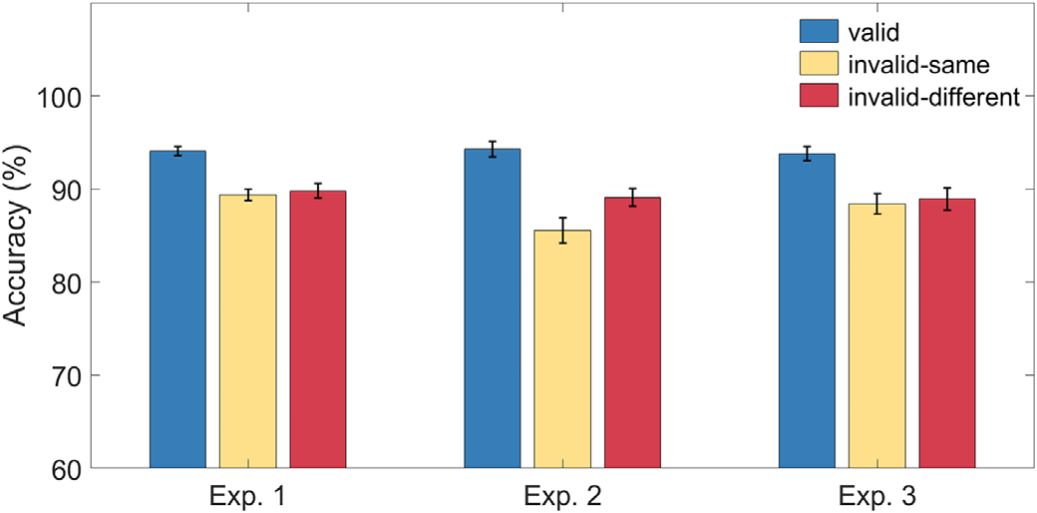
Mean accuracy and standard errors (SEM) for valid (*blue*), invalid-same (*yellow*), and invalid-different condition (*red*) within each experiment.

High accuracy was shown for each experiment (Experiment 1: *M* = 93%, *SD* = 3.2; Experiment 2: *M* = 91%, *SD* = 5.1; Experiment 3: *M* = 90%, *SD* = 5.2), and for each condition within each experiment (Figure 2). One-way ANOVA were conducted on accuracy data for each experiment. The results showed significant main effects of condition (valid, invalid-same, invalid-different) for Experiment 1, *F* (1, 39) = 62.43, *p* < 0.001, η_p_^2^ = .62; Experiment 2, *F* (1, 40) = 69.24, *p* < 0.001, η_p_^2^ = .63; and Experiment 3, *F* (1, 39) = 35.16, *p* < 0.001, η_p_^2^ = .47.

Further *t*-tests revealed that accuracy for valid conditions was significantly higher than invalid conditions for all three experiments, *ts* > 5, *ps* < 0.001. In Experiment 2, accuracy was significantly higher for invalid-different conditions than for invalid-same conditions, *t* (40) = −4.28*, p* < 0.001. In Experiments 1 and 3, there were no differences in accuracy between invalid-same and invalid-different conditions (Experiment 1, *t* (39) = −0.73, *p* = 0.47; Experiment 3, *t* (39) = −0.49, *p* = 0.63).

### RT data

Experiment 1 investigated space- and object-based effects with 80% cued validity. The ANOVA showed significant main effects of condition, *F* (2, 78) = 73.17, *p* < 0.001, η_p_^2^ = .65. Participants responded faster for valid condition (*M* = 536, *SD* = 63) than invalid-same condition (*M* = 622, *SD* = 75, *t* (39) = −9.68, *p* < 0.001), or invalid-different condition (*M* = 638, *SD* = 88, *t* (39) = −8.61, *p* < 0.001). Responses were faster for invalid-same condition than invalid-different condition, *t* (39) = −2.98, *p* = 0.005. RTs significantly differ between cue-to-target intervals, *F* (30, 1170) = 2.63, *p* = 0.006, η_p_^2^ = .06. No significant interaction was found between condition and cue-to-target interval, *F* (60, 2340) = .98, *p* = 0.46.

**Figure 3.**
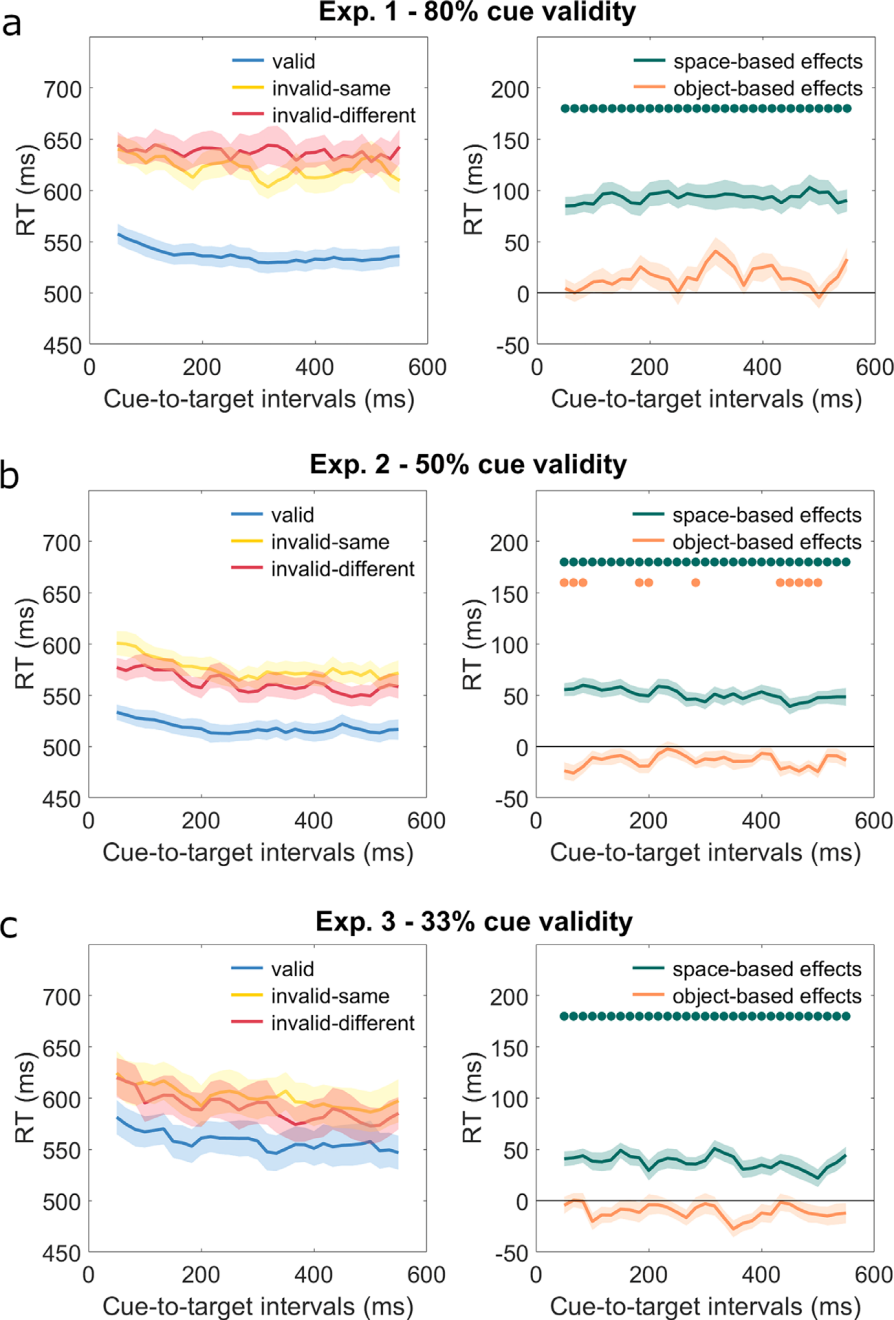
Mean RT ± SEM for three conditions (left) and for space- and object-based effects (*right*) in Experiment 1 (a), Experiment 2 (b), and Experiment 3 (c). *Green dots* in the upper part of the figures on the right indicate cue-to-target intervals with significant space-based effect (*p* < 0.05), and *orange dots* indicate cue-to-target intervals with significant negative object-based effects (*p* < 0.05). There are no intervals with significant positive object-based effects.

Further *t*-tests investigated overall space- and object-based effects at each cue-to-target interval. Significant space-based effects were found in all of the cue-to-target intervals, whereas no significant object-based effects were found (Figure 3a).

Experiment 2 investigated space- and object-based effects with a reduced cue validity (50%) and increased probability that a target would appear at uncued locations (25%). The ANOVA showed significant main effects of condition, *F* (2, 80) = 63.97, *p* < 0.001, η_p_^2^ = .62. Responses were faster for valid condition (*M* = 518, *SD* = 54) than for invalid-same condition (*M* = 576, *SD* = 63, *t* (40) = −10.52, *p* < 0.001) or for invalid-different condition (*M* = 562, *SD* = 62, *t* (40) = −6.49, *p* < 0.001). For invalid conditions, RTs were shorter for invalid-different condition than for invalid-same condition, *t* (40) = −4.52, *p* < 0.001. RTs significantly varied between cue-to-target intervals, *F* (30, 1200) = 8.02, *p* < 0.001, η_p_^2^ = .17. There was no interaction of condition and cue-to-target interval, *F* (60, 2400) = 1.18, *p* = 0.28. To investigate the prevalence of space- and object-based effects at each cue-to-target interval, we conducted *t*-tests, which showed that space-based effects were significant across all cue-to-target intervals, but not object-based effects. However, several cue-to-target intervals showed significant negative object-based effects (Figure 3b), which means participants responded faster in trials where targets appeared at different-object location (invalid-different condition) than at same-object location (invalid-same condition).

Experiment 3 investigated space- and object-based effects with targets appearing at cued, same-object, and different-object locations with equal probability. The ANOVA again showed significant main effects of condition, *F* (2, 78) = 27.18, *p* < 0.001, η_p_^2^ = .41. Among three conditions, RTs were shorter for valid condition (*M* = 558, *SD* = 106) than for invalid-different condition (*M* = 591, *SD* = 114, *t* (39) = −4.66, *p* < 0.001) or for invalid-same condition (*M* = 601, *SD* = 122, *t* (39) = −6.88, *p* < 0.001). RTs for invalid-different condition were shorter than for invalid-same condition, *t* (39) = −2.20, *p* = 0.03. RTs significantly varied between cue-to-target interval, *F* (30, 1170) = 10.16, *p* < 0.001, η_p_^2^ = .21. There was no significant interaction between condition and cue-to-target intervals, *F* (60, 2340) = 0.99, *p* = 0.45. To investigate the prevalence of space- and object-based effects at each cue-to-target interval, we conducted *t*-tests, which showed significant space-based effects across all the cue-to-target intervals but not object-based effects (Figure 3c).

**Figure 4.**
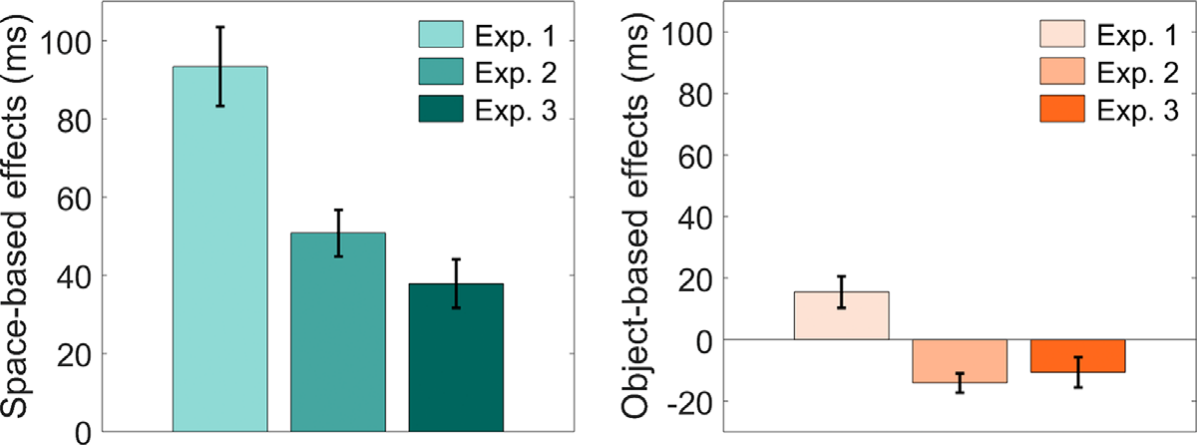
Space- (*left*) and object-based effects (*right*) in response time across all experiments. *Error bars*: represent the standard error of the mean.

Figure 4 shows magnitudes for space- and object-based effects for three experiments. A one-way ANOVA with experiment as a between subject factor found significant differences in magnitudes of space-based effects across experiments, *F* (2, 118) = 14.46, *p* < 0.001, η_p_^2^ = .20. Further *t*-tests revealed that space-based effects in Experiment 1 (*M* = 93, *SD* = 64) were significantly larger than effects in Experiment 2 (*M* = 51, *SD* = 38, *t* (79) = 3.66, *p* < 0.001) and Experiment 3 (*M* = 37, *SD* = 39, *t* (78) = 4.70, *p* < 0.001). Although space-based effects were larger in Experiment 2 than in Experiment 3, this difference did not reach significance, *t* (79) = 1.51, *p* = 0.14.

For object-based effects, a one-way ANOVA found that magnitudes significantly differ between experiments, *F* (2, 118) = 13.11, *p* < 0.001, η_p_^2^ = .18. Object-based effects in Experiment 1 (*M* = 16, *SD* = 33) were larger than effects in Experiment 2 (*M* = −14, *SD* = 20, *t* (79) = 4.91, *p* < 0.001) and Experiment 3 (*M* = −11, *SD* = 31, *t* (78) = 3.68, *p* < 0.001). No significant differences in the magnitudes of object-based effects were found between Experiment 2 and Experiment 3, *t* (79) = −.59, *p* = 0.56.

### Bootstrapping of individual participants

**Figure 5.**
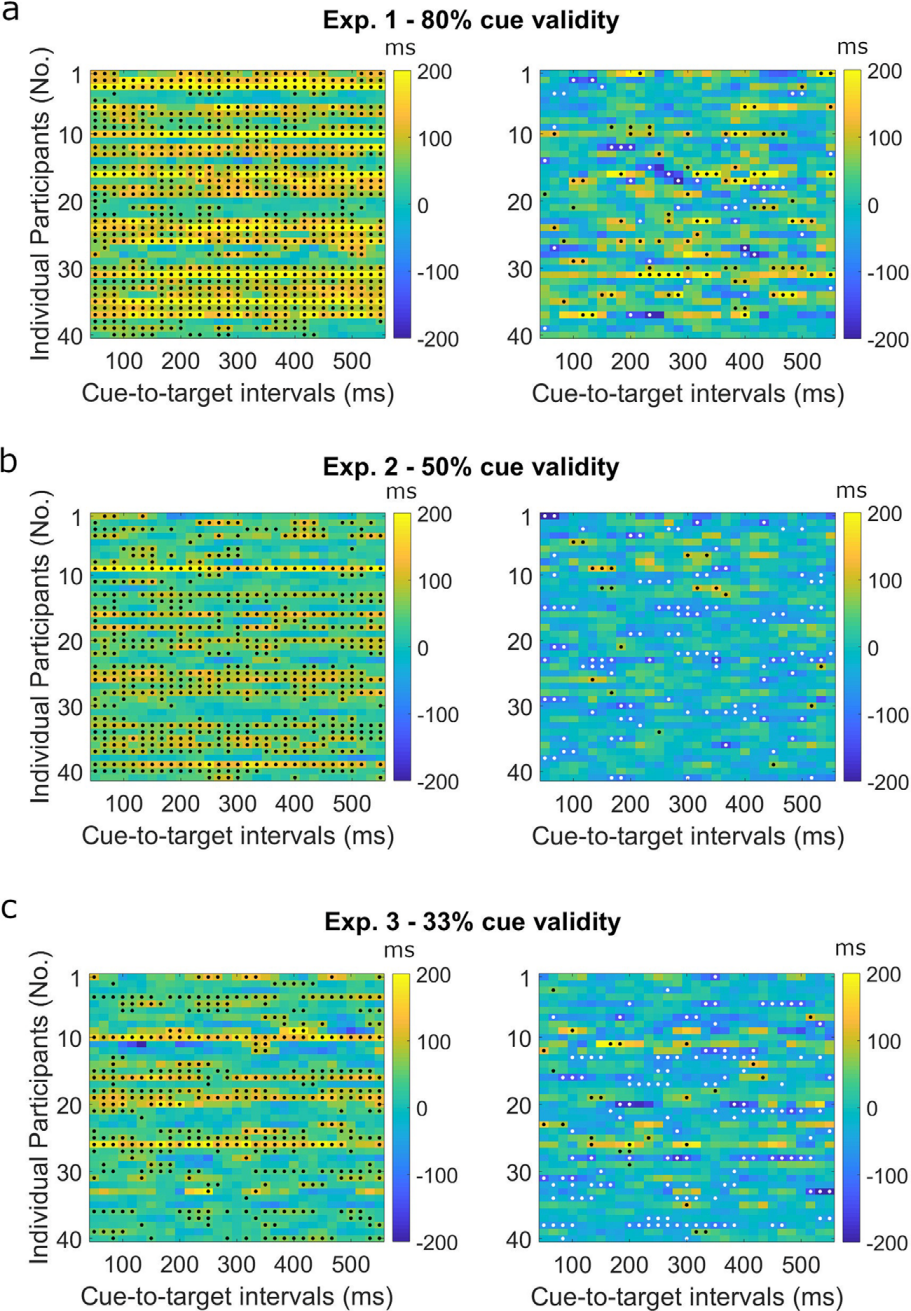
Bootstrapped space- (left) and object-based effects (right) of each participant in Experiment 1 (a), Experiment 2 (b), and Experiment 3 (c). *Black dots* indicate significance at *p* < 0.05 for space-based effects (left) and object-based effects (right). *White dots* indicate significant negative object-based effects *(p* < 0.05).

**Figure 6.**
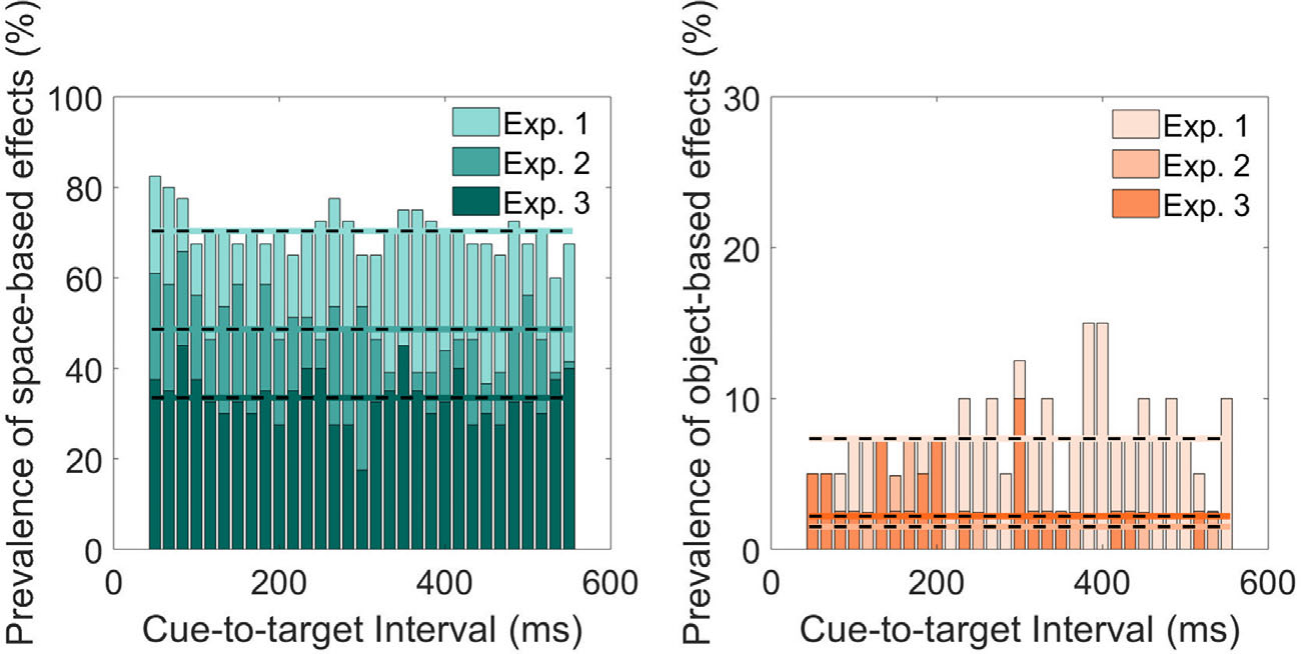
Percentage of participants showing significant space- (*left*) and object-based effects (*right*) for each cue-to-target interval in Experiment 1 (80% cue validity), Experiment 2 (50% cue validity), and Experiment 3 (33% cue validity). The *horizontal dashed lines* represent the prevalence of space- and object-based effects as expressed in the average percentage of participants showing space- (*left*) and object-based effects (*right*).

Results of the bootstrap analysis for space- and object-based effects are summarized in Figure 5 and Figure 6. In Experiment 1, all participants showed significant space-based effects for at least one cue-to-target interval. Object-based effects were less prevalent, with 65% of the participants showing significant object-based effects for at least one cue-to-target interval. Sixty percent of participants showed significant negative object-based effects for at least one cue-to-target interval. Within each cue-to-target interval, around 70% of participants showed significant space-based effects, only around 7% of participants showed significant object-based effects.

In Experiment 2, with cue validity reduced to 50%, space-based effects were less pronounced than the effects in Experiment 1 but were still more prevalent than the object-based effects. Ninety-five percent of participants showed significant space-based effects for at least one cue-to-target interval, whereas this proportion for object-based effects was only at 29%. Most participants (71%) showed negative object-based effects. Within each cue-to-target interval, around 49% of the participants showed space-based effects, significant object-based effects were only found in several participants (2%).

In Experiment 3, cue validity further decreased to 33%. Ninety-five percent of participants showed significant space-based effects for at least one cue-to-target interval, whereas 43% of participants showed significant object-based effects for at least one cue-to-target interval. Sixty percent of participants showed negative object-based effects. Although space-based effects were found in most participants, space-based effects were not stable across cue-to-target intervals. Within each cue-to-target interval, only around 33% of the participants showed space-based effects. Object-based effects were as sparse as in Experiment 2, and only shown in around 2% of the participants.

### Exploratory analysis

Observed object-based effects in Experiment 2 and 3 resemble effects of inhibition-of-return (IOR). It has been shown that facilitatory and inhibitory cueing effects depend on the cue-to-target stimulus onset asynchrony (SOA). Facilitatory effects has been found with short SOAs (within 300 ms) and inhibitory effects has been found with long SOAs (e.g., Klein & Ivanoff, 2008; List & Robertson, 2007). To assess negative object-based effects within the context of the existing literature on IOR, we have added an additional analysis in which we compared whether space- and object-based effects differed between short and long cue-to-target intervals. In accordance with the previous literature, we separated cue-to-target intervals (CTIs) into those shorter than 300 ms (short CTIs), and longer than 300 ms (long CTIs). We then conducted a three-way ANOVA, with attentional cueing effect (space- and object-based effects), CTI (short, long) as within-subject factors, and cue validity (80%, 50%, 33%) as a between-subject factor. The main effect of CTI was not significant (*p* = 0.77, η_p_^2^ < .01). Interaction effects of CTI × attentional effects (*p* = 0.22, η_p_^2^ = .01) and CTI × attentional effects × cue validity (*p* = 0.84, η_p_^2^ < .01) were not significant. These results showed no significant differences between space- and object-based effects for shorter and longer CTIs.

## Discussion

Over three experiments on the mechanisms underlying attentional selection, we manipulated cue validity from 80% (Experiment 1) to 50% (Experiment 2) and 33% (Experiment 3). We investigated whether and how cue validity affects the temporal occurrence of space- and object-based attentional selection on both group and individual level on the basis of response times. On the group level, space-based effects were more robust than object-based effects for all three experiments and all cue-to-target intervals. However, when reducing cue validity from 80% to 50% and 33%, the magnitude of space-based effects decreased significantly. With 80% cue validity, significant object-based effects were observed. As cue validity decreased from 80% to 50% or 33%, object-based effects became smaller or even negative, such that participants performed better on invalid-different trials than on invalid-same trials (different-object advantage). On the individual level, space-based effects were more prevalent than object-based effects in all three experiments. With decreased cue validity, the prevalence of space-based effects dropped profoundly. With a cue validity of 33%, significant space-based effects were observed in a small percentage of participants within a single cue-to-target interval. Object-based effects were not prevalent in all three experiments, because significant object-based effects were only found in a minority of participants for each cue-to-target interval.

Our findings are compatible with previous studies that found space-based effects to be stronger (e.g., Egly et al., 1994; Hecht & Vecera, 2007; Moore et al., 1998) and more prevalent than object-based effects (e.g., Pilz et al., 2012; Lou et al., 2020). Manipulating cue-to-target intervals from 50 to 600 ms, Lou et al. (2020) found that the cue-to-target intervals where object-based effects occur differed largely between individuals. Similar findings were observed in the current study. Although object-based effects were significant on the group level, they were less prevalent than space-based effects. In addition, individuals differed in the timepoint at which object-based effects occurred, with most participants showing object-based effects only at a few timepoints. These findings support the idea that there are individual differences in the temporal occurrence of object-based effects.

Our results are also in line with studies showing significant object-based effects using an varying cue-to-target interval (e.g., Fiebelkorn et al., 2013; Helfrich et al., 2018; Peters et al., 2020). Studies with fixed cue-to-target intervals were inconsistent regarding object-based effects, because some found significant effects (e.g., Egly et al., 1994; Hecht & Vecera, 2007; Moore et al., 1998), whereas others did not (Macquistan, 1997). Because object-based effects seem to differ largely across individuals in their temporal occurrence, it is likely that studies using constant cue-to-target intervals may have captured object-based effects only for a limited number of participants (Pilz et al., 2012), resulting in inconsistent findings about object-based effects.

Spatial probability has been found to effectively guide attention (Druker & Anderson, 2010; Geng & Behrmann, 2005; Jiang et al., 2013; Kabata & Matsumoto, 2012). It has been shown that detecting targets in high-probability locations is faster than detecting targets in low-probability locations (e.g., Geng & Behrmann, 2005). Our results confirm these findings for a cue validity of 80%, but highlight a significant decrease in the magnitude and prevalence of space-based effects when cue validity is reduced to at or below 50%. This is consistent with findings showing that space-based effects became smaller by reducing cue validity (e.g., Chou & Yeh, 2018; He, Fan, Zhou, & Chen, 2004). These results also support our hypothesis that probability information affects attention towards locations with the highest target probability. That is, high cue validity may facilitate attentional allocation to the cued locations, resulting in strong and consistent space-based effects.

Surprisingly, we found significant negative object-based effects on group level (Experiment 2 and 3) and at the individual level (all three experiments). Importantly, negative object-based effects seem to be more prevalent when cue validity dropped from 80% to 50% or 33% (Figure 5). Space-based effects, in contrast, did not become negative. This might be related to different representations underlying space- and object-based effects. It has been shown that representations of locations in space and objects affect attentional selection, leading to space- and object-based effects (Possin, Filoteo, Song, & Salmon, 2009; Reppa, Schmidt, & Leek, 2012). It has also been shown that spatial representations are prioritized over object representations. When targets are biased toward invalid different-object locations, object representations fail to guide attention, whereas spatial representations remain effective (Nah & Shomstein, 2020). In our study, the negative object-based effects were likely a result of weakened object representations due to reduced cue validity. Spatial representations were also weakened given that magnitudes greatly decreased, but still remained effective in guiding attention.

The negative object-based effects observed in our study strongly resemble effects of IOR. IOR describes situations in which target detection is slower at cued compared to uncued locations (Posner & Cohen, 1984). In a typical IOR experiment, target detection is facilitated at the cued location when the cue-to-target SOA is below 300 ms. However, when it is longer than 300 ms, responses to the target are slower at the cued location relative to an uncued location, demonstrating location-based IOR. It has been suggested that IOR prevents repeated sampling of locations that have already been searched (Klein, 1988). IOR has also been shown for object-based paradigms (Chou & Yeh, 2018; Jordan & Tipper, 1999; Leek, Reppa, & Tipper, 2003; List & Robertson, 2007; Possin et al., 2009). However, it is difficult to directly compare our results to the previously shown effects. First, in typical IOR experiments, an unpredictive cue is used, such that target probability is equal across locations (e.g., Jordan & Tipper, 1999; List & Robertson, 2007), which is not the case for our study, in which target probability was systematically changed across experiments. More importantly, however, negative object-based effects in our experiment were shown across all SOAs, whereas IOR is usually found for longer SOAs only. Therefore it is questionable whether the functional mechanism, to prevent revisiting already searched locations, is the same. To assess the more direct link between negative object-based effects and IOR, we have conducted an additional analysis to assess differences between object-based effects for short and long cue-to-target intervals. This analysis did not reveal any systematic differences.

The effect of the peripheral cues used in our study involved both exogenous/reflexive and endogenous/voluntary orienting of visual attention. It has been shown that the exogenous orienting is triggered by the physical properties of the cue and is little affected by cue validity. Cue validity, however, influences endogenous orientation (Müller and Rabbitt, 1989; Müller & Findlay, 1988). Therefore, with a cue validity of 33% in Experiment 3, the endogenous orienting should be minimized because it was uninformative of the target location. Our results showed space-based effects remained significant but reduced in magnitude with decreasing cue validity. Moreover, as cue validity reduced, same-object advantage shifted to different-object advantage. These results suggest that space- and object-based effects may be related to both endogenous and exogenous orienting.

Different to previous studies, our study only used horizontal rectangles compared to the classical experiment with both horizontal and vertical rectangles(e.g., Chou & Yeh, 2018; Egly et al., 1994; Nah & Shomstein, 2020; Pilz et al., 2012). This choice was made given the emphasis on temporal aspects of object-based attention and the duration of the experiment. Not many studies include object-orientation as a factor in their analysis. Comparing performance in object-based attention tasks, studies have found similar object-based effects for vertical and horizontal rectangles (e.g., Fiebelkorn et al., 2013; Nah & Shomstein, 2020), whereas others showed an advantage for horizontal rectangles (Barnas & Greenberg, 2016; Hein et al., 2017; Pilz et al., 2012), which has been attributed to an advantage in allocating attention horizontally. Chen and Cave (2019) even found that object-based effects could be entirely explained by a horizontal advantage. To assess whether including both object orientations would have made a difference to the results reported here, we conducted a follow-up experiment, similar to Experiment 2 with 50% cue validity, which included both horizontal and vertical orientation. The experiment is included in the supplementary information. Results were similar for horizontally and vertically oriented rectangles, and the pattern of results did not differ substantially from the experiment with horizontal rectangles only. These results indicate that including both object orientations in our experiments would not have changed the pattern of results significantly.

It has to be noted that the current study was conducted online. In laboratory experiments, experimental settings and the environment, in which an experiment is conducted, are usually controlled consistently across participants to avoid effects from confounding variables. Compared with lab experiments, environments of online experiments are less controlled and variables such as the lighting of the room, stimulus luminance and size (relating to the size of the screen, and the distance from the screen) may vary across participants. The current findings, however, are largely in line with prior lab studies, such as significant attentional cueing effects (e.g., Egly et al., 1994; Nah & Shomstein, 2020; Pilz et al., 2012) and large individual differences in object-based effects (e.g., Pilz et al., 2012; Lou et al., 2020). However, since the online environment was less controlled, we cannot rule out that this may have affected our results (e.g., more participants were excluded because of their poor performance).

In conclusion, the current study adds to the evidence for large individual differences in the temporal occurrence of attentional selection. With a reduction in cue validity, space-based effects became smaller and less stable across time. Object-based effects remained small and tended to be negative. The less controlled online experiment settings may have influenced the current results, and this needs to be further investigated. Overall, our results suggested that spatial probability and individual variations should be considered in developing theories of visual attention.

## Supplementary Material

Supplement 1

## Footnotes

1 Given the emphasis on temporal aspects of object-based attention and the duration of the experiment, the current study only included horizontal objects rather than randomly choosing from vertical or horizontal object orientations. Not many studies include object-orientation as a factor in their analysis. Comparing performance in object-based attention tasks, studies have found similar object-based effects for vertical and horizontal rectangles (e.g., Fiebelkorn et al., 2013; Nah & Shomstein, 2020), whereas others showed an advantage for horizontal rectangles (Barnas & Greenberg, 2016; Hein et al., 2017; Pilz et al., 2012), which has been attributed to an advantage in allocating attention horizontally (also see Chen & Cave, 2019). As a follow-up to Experiment 2, we conducted an experiment with 50% cue validity, which included both horizontal and vertical orientation (see Supplementary Information). Results were similar for horizontally and vertically oriented rectangles. Based on these results, including both orientations might not change the observed results in the current study.
