# Supplementary material for: Effects of cue validity on attentional selection
Source: J Vis. 2022 Jul 26;22(8):15. doi: 10.1167/jov.22.8.15 (PMC9339692; doi:10.1167/jov.22.8.15)
Supplement: Supplement 1 [file jovi-22-8-15_s001.pdf]

Supplementary Information

To examine the effect of rectangle orientation on object-based effects, we conducted an additional experiment with 50% cue validity, in which we included both horizontal and vertical rectangles.

The additional experiment was created using PsychoPy/PsychoJS, v2020.1.3 (Peirce et al., 2019), and was conducted online through Pavlovia (www.pavlovia.org). The size of the stimuli and the experimental procedures used in this experiment were the same as in the original study. Instead of using only horizontal rectangles, the stimulus display in the additional experiment was modified to use horizontal or vertical rectangles. For each trial, the orientation of rectangles was chosen randomly so that participants did not know the orientation before the trial began. The duration of the experiment remained the same as Experiment 2. A total of 816 trials was collected, of which 50% were valid, 25% were invalid-same trials, and 25% were invalid-different trials. A practice block was conducted prior to the experiment to familiarize participants with the procedure.

We collected data from 41 participants (18–27 years, M = 22.2, SD = 2.4, 22 males), using the same analysis as in Experiment 2. Similar to results of Experiment 2, significant space-based effects were shown across all the cue-to-target intervals (*p*s < 0.05, FDR corrected), whereas no significant object-based effects (*p*s > 0.05, FDR corrected, Figure *S*1). Within each cue-to-target interval, around 35% of participants showed space-based effects, significant object-based effects were only found in 5% of participants (Figure *S*2, Figure *S*3).

To examine the effects of orientation, an Orientation (horizontal, vertical) x Cueing effects (space-based effects, object-based effects) ANOVA was conducted on reaction times. We did not find significant effect of orientation, $F\,(1,40) = 0.30$, $p = 0.59$. Main effect of cueing effects was significant, as space-based effects (34 $\pm$ 8 ms) were significantly larger than object-based effects (4$\pm$5 ms), $F\,(1,40) = 36.67$, $p < 0.01$, $\eta_p^2 = .48$. There also was an interaction between orientation and cueing effects, $F\,(1,40) = 5.59$, $p = 0.02$, $\eta_p^2 = .12$. Post-hoc tests found no significant difference between horizontal and vertical orientation in space- (t (40) = -1.85, p = 0.07) or object-based effects (t (40) = 1.49, p = 0.14). The interaction was driven by significant differences between space- object-based effects across orientations (see Figure S4). Mean RT and standard deviation of space- and object-based effects for two orientations are shown in Table S1. Figure S5 shows RT patterns for the two orientations separately, which are very similar: space-based effects are stronger than object-based effects, and object-based effects are small and did not reach significance. As horizontal and vertical orientations have

shown similar patterns for space- and object-based effects, including both orientations might not change the observed results in the current study. The observed effects of cue validity on object-based effects in the present study seem to reflect the impact of cue validity on object-based guidance of attention rather than on attentional benefits on the horizontal meridian.

Table *S*1. Mean and standard deviation of space- and object-based effects for horizontal and vertical orientation

orientation	cueing effects	Mean	SD	N
horizontal	space-based effects	33.63	29.24	41
	object-based effects	7.1	19.75	41
vertical	space-based effects	36.3	27.97	41
	object-based effects	0.51	22.09	41

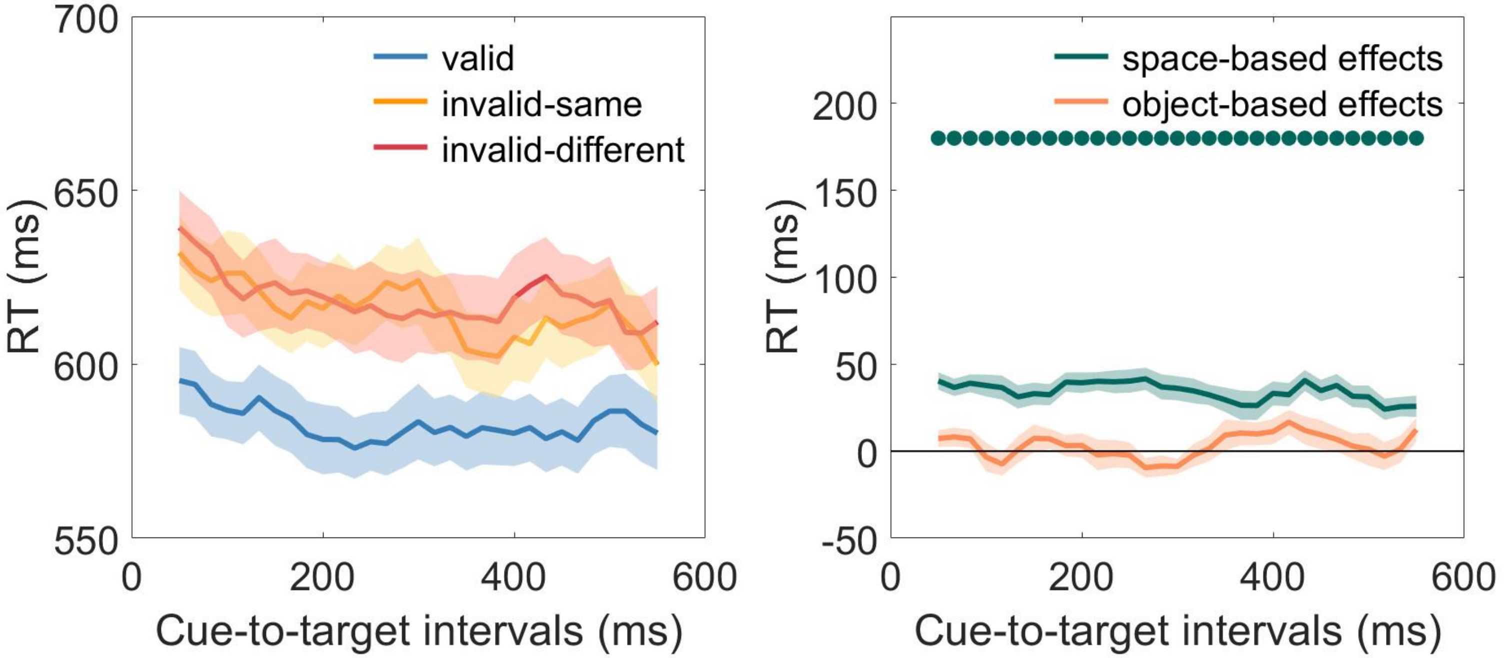

*Figure S*1. Mean RT ± SEM for three conditions (left) and for space- and object-based effects (right).
Green dots in the upper part of the figures on the right indicate cue-to-target intervals with significant space-based effect (*p* <.05, FDR corrected). There are no intervals with significant object-based effects.

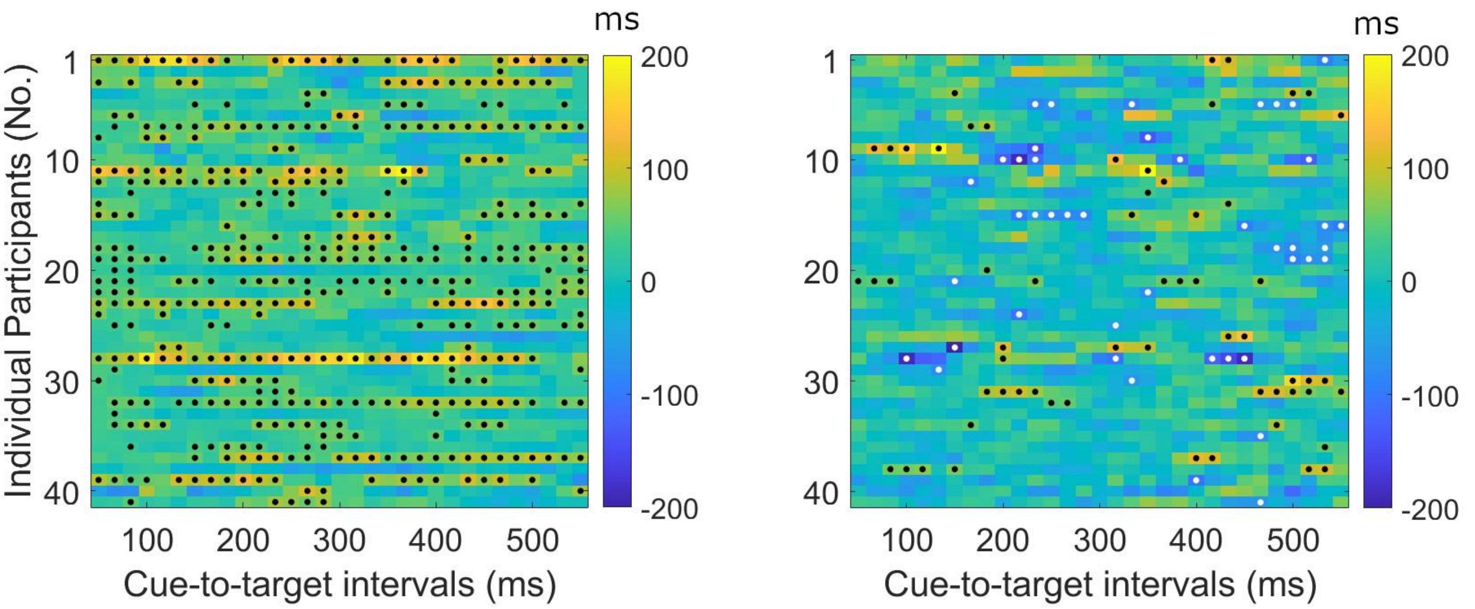

Figure S2. Bootstrapped space- (left) and object-based effects (right) of each participant.
Black dots indicate significance at p <.05 for space-based effects (left) and object-based effects (right).
White dots indicate significant negative object-based effects (p <.05).

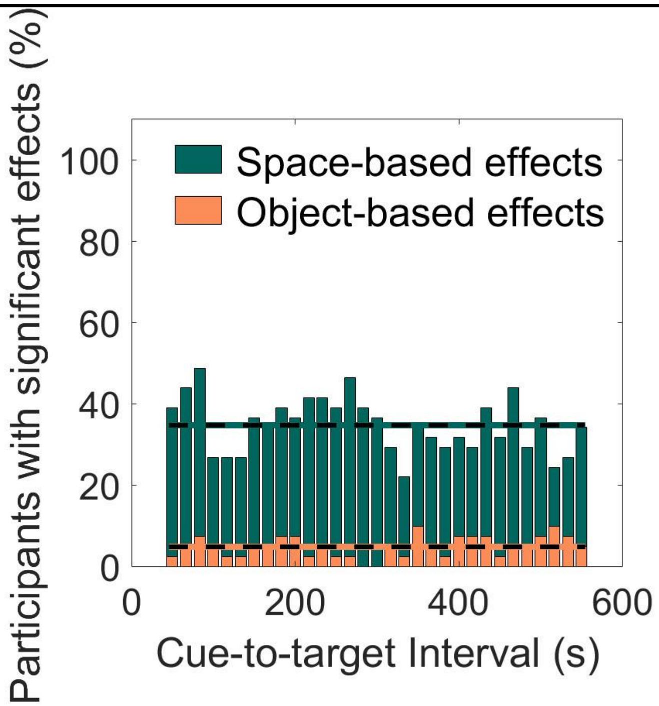

*Figure S*3. Percentage of participants showing significant space- and object-based effects for each cue-to-target interval. The horizontal dashed lines represent the prevalence of space- and object-based effects as expressed in the average percentage of participants showing space- (green) and object-based effects (orange).

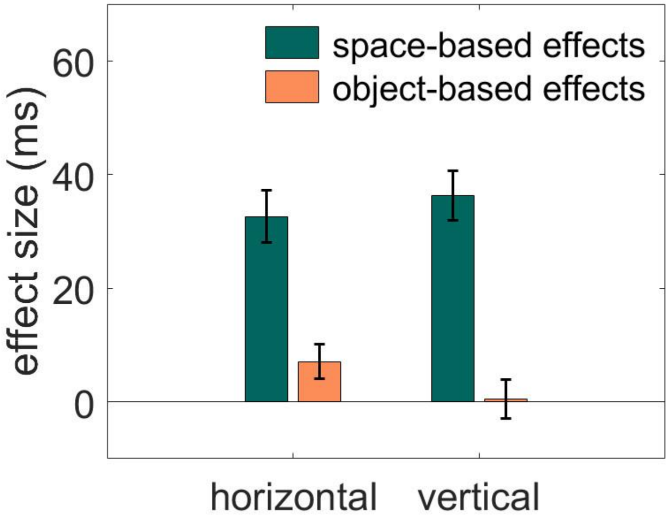

*Figure S*4. Space- and object-based effects (*M*±SEM) for horizontal (left) and vertical orientation (right).
* Indicates significance at $p < 0.05$.

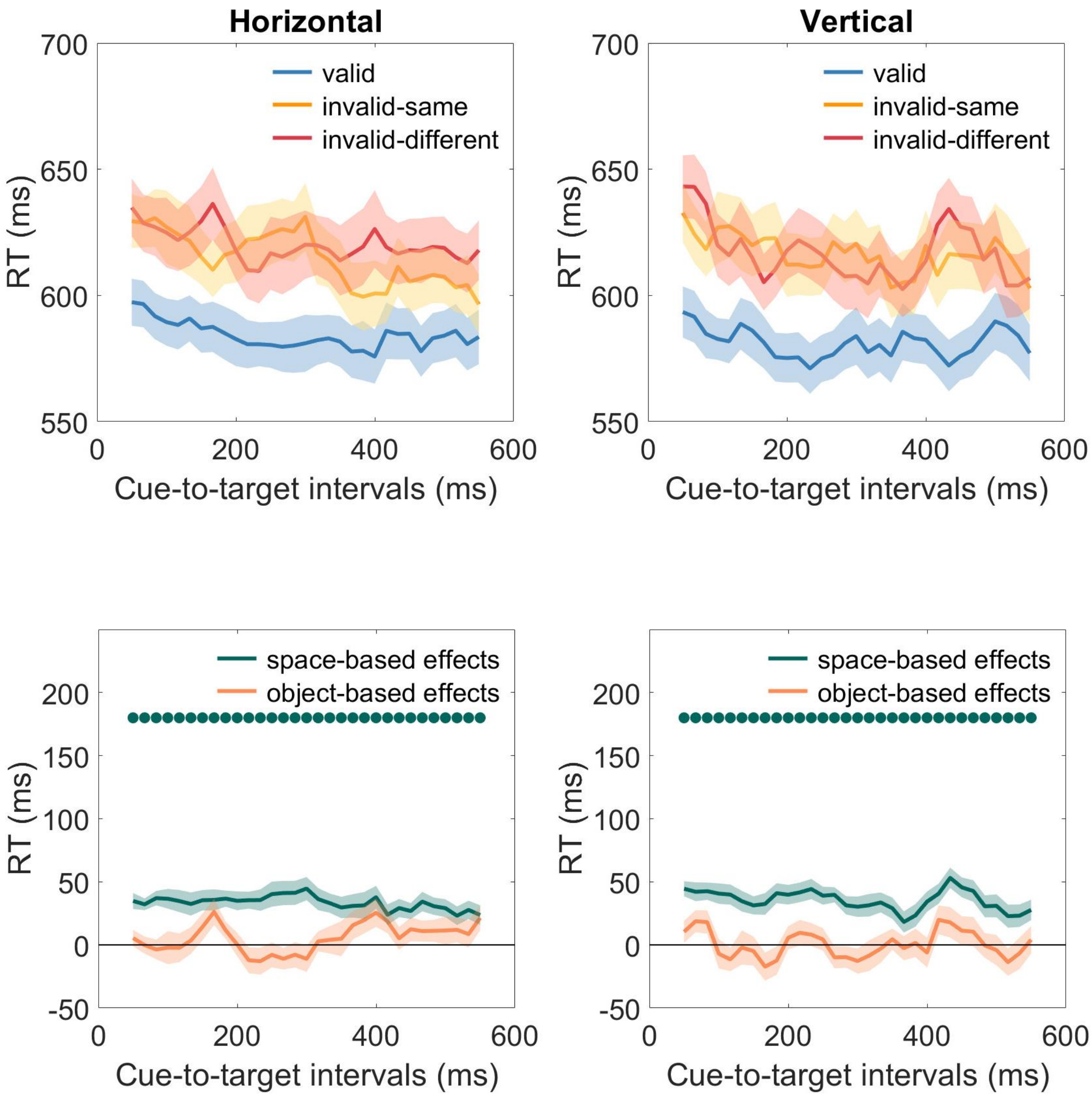

*Figure S*5. RT results for horizontal (left) and vertical orientation (right).
Green dots indicate cue-to-target intervals with significant space-based effect (p <.05, FDR corrected).
